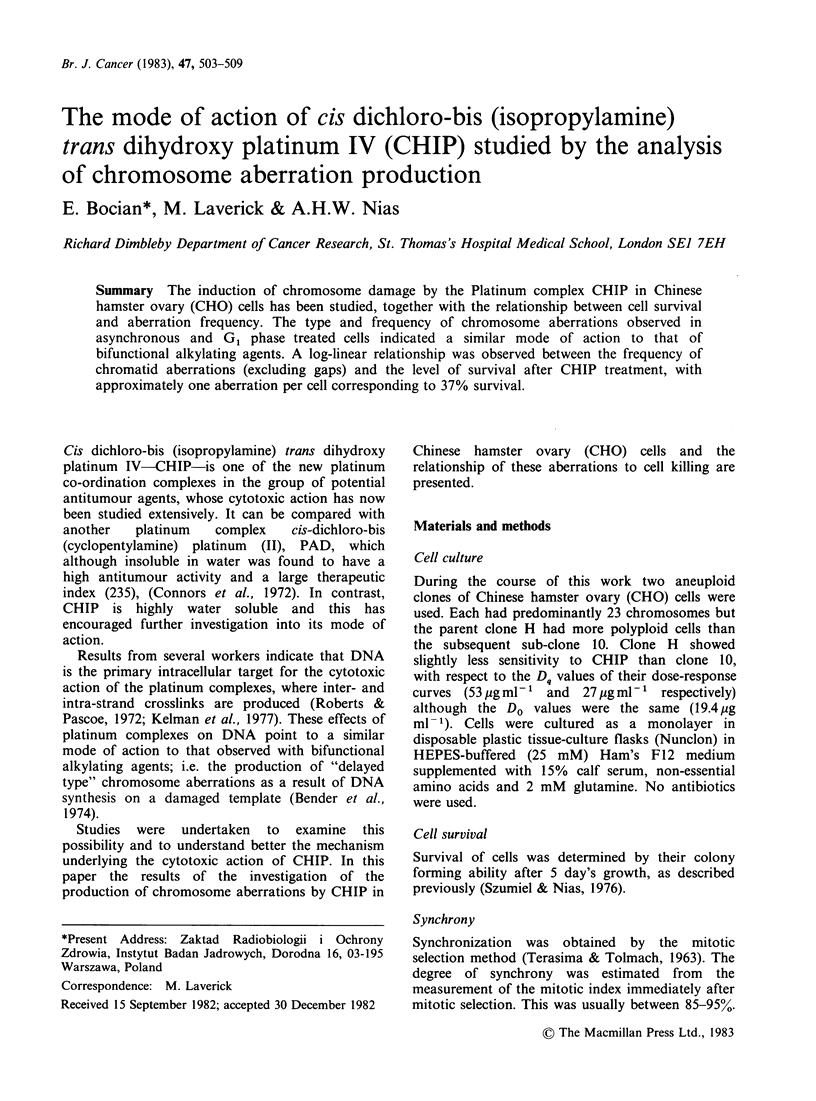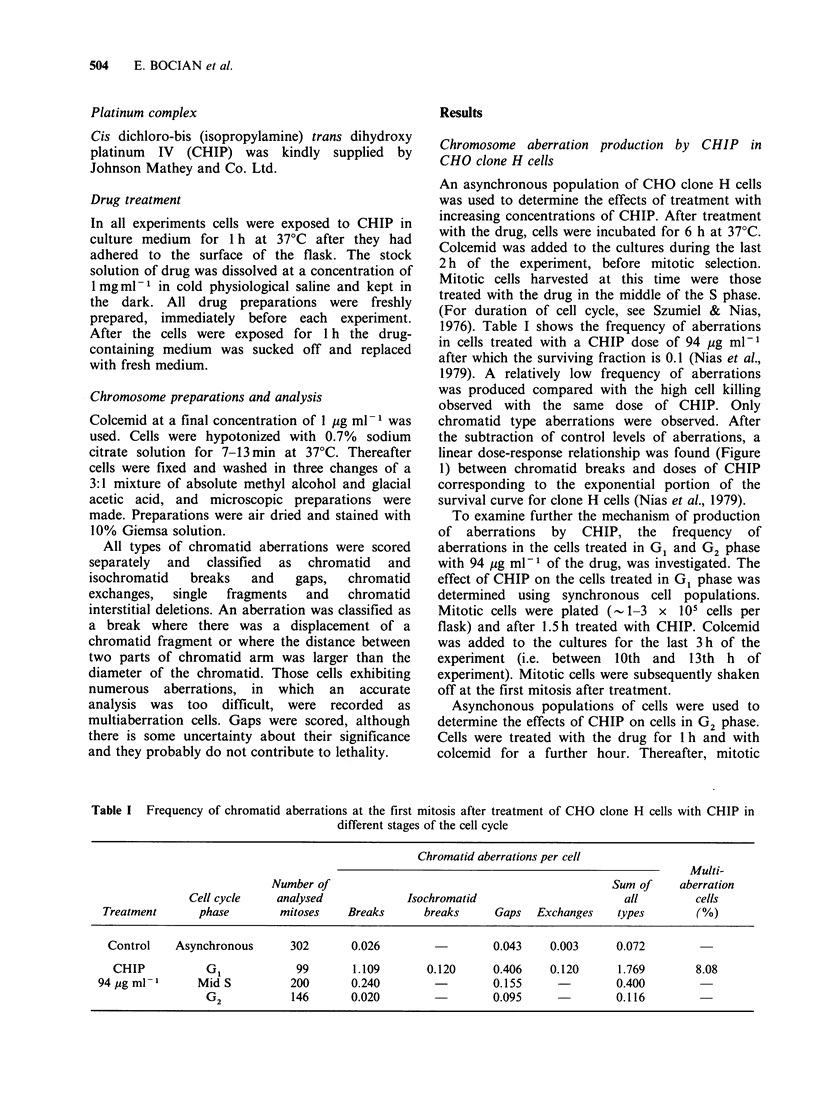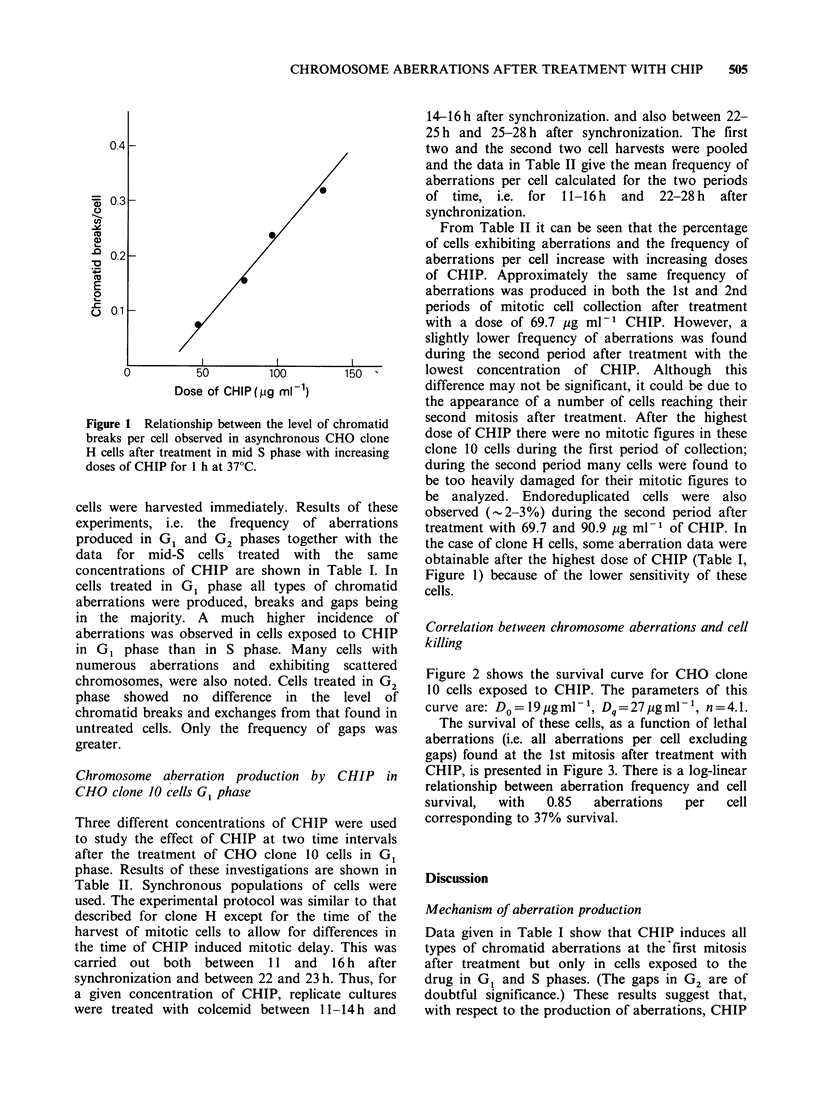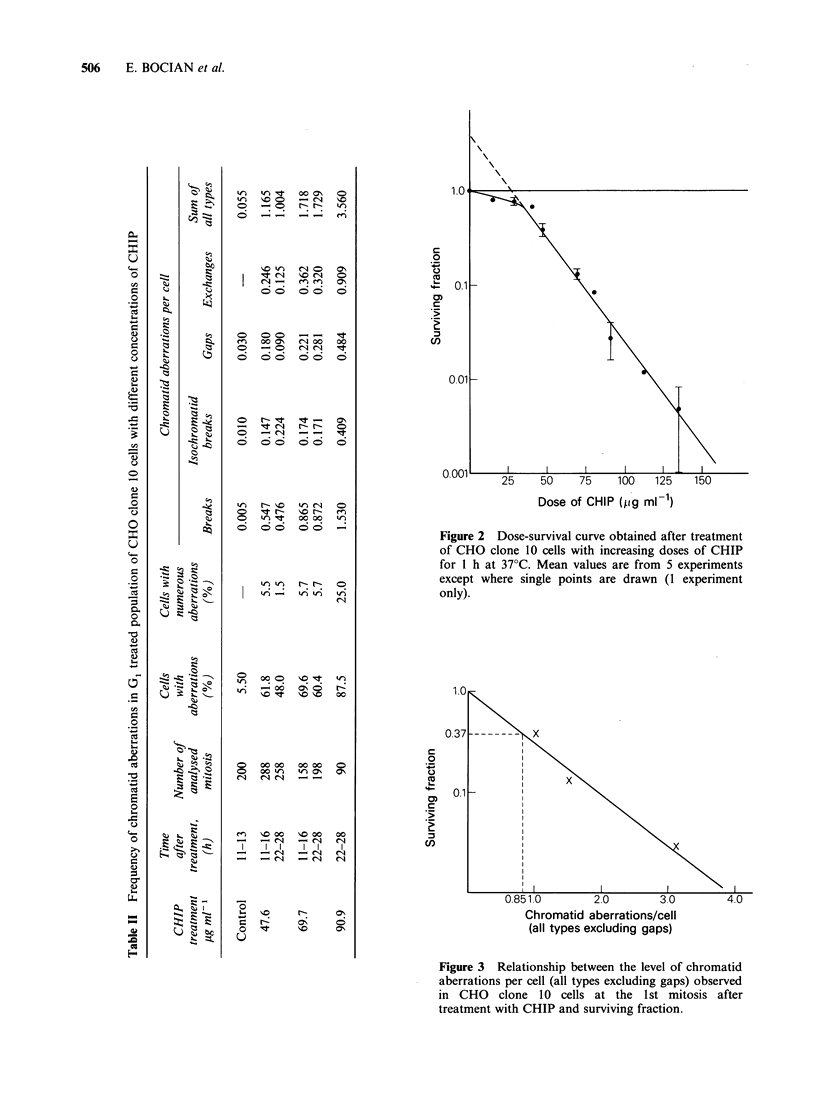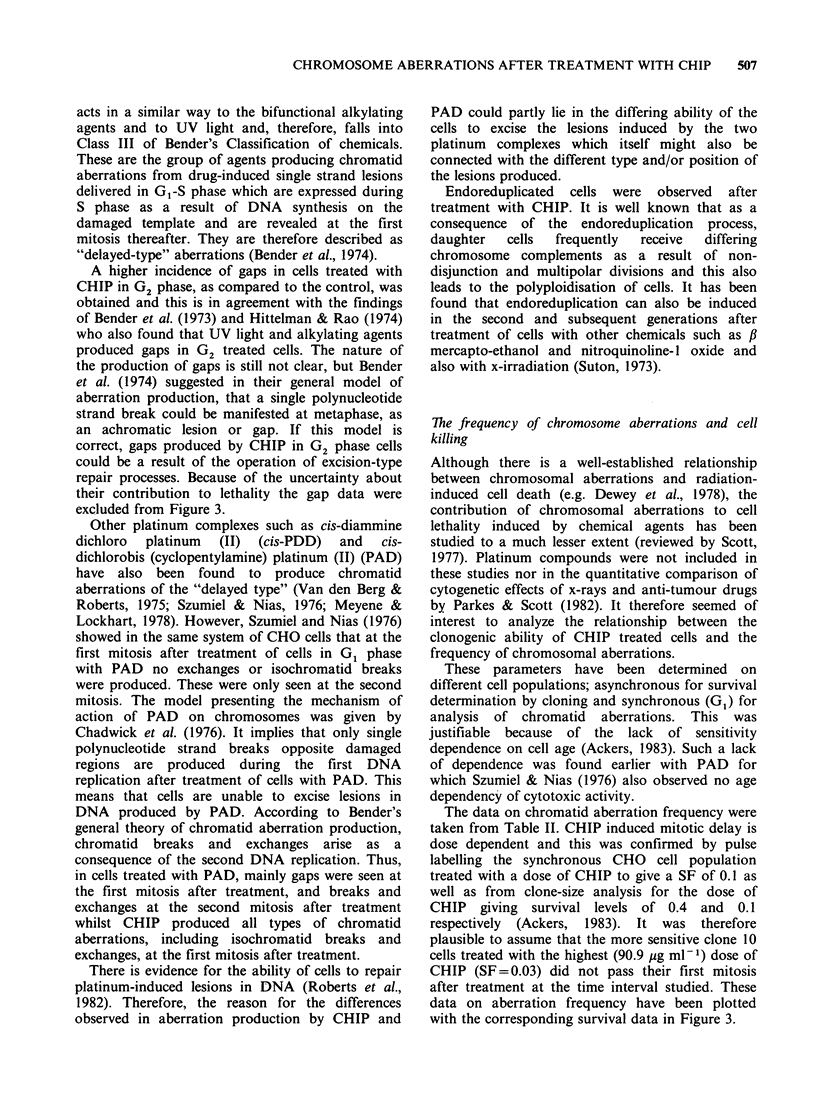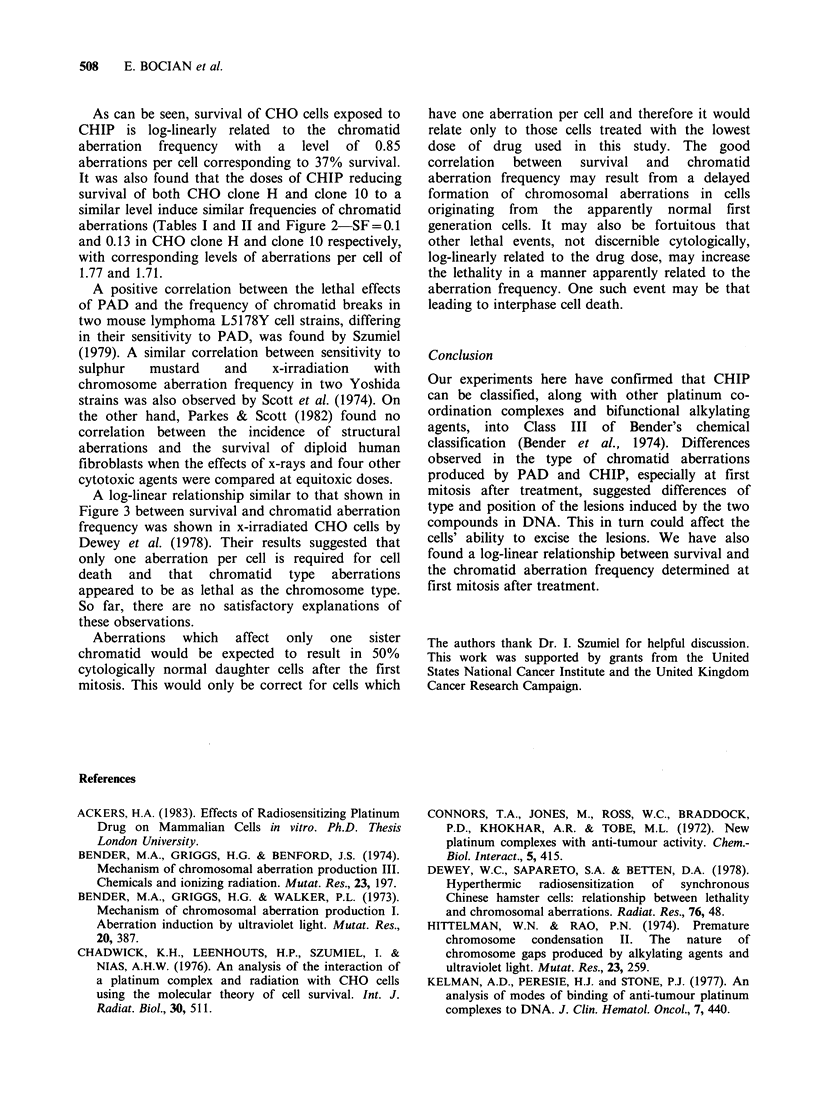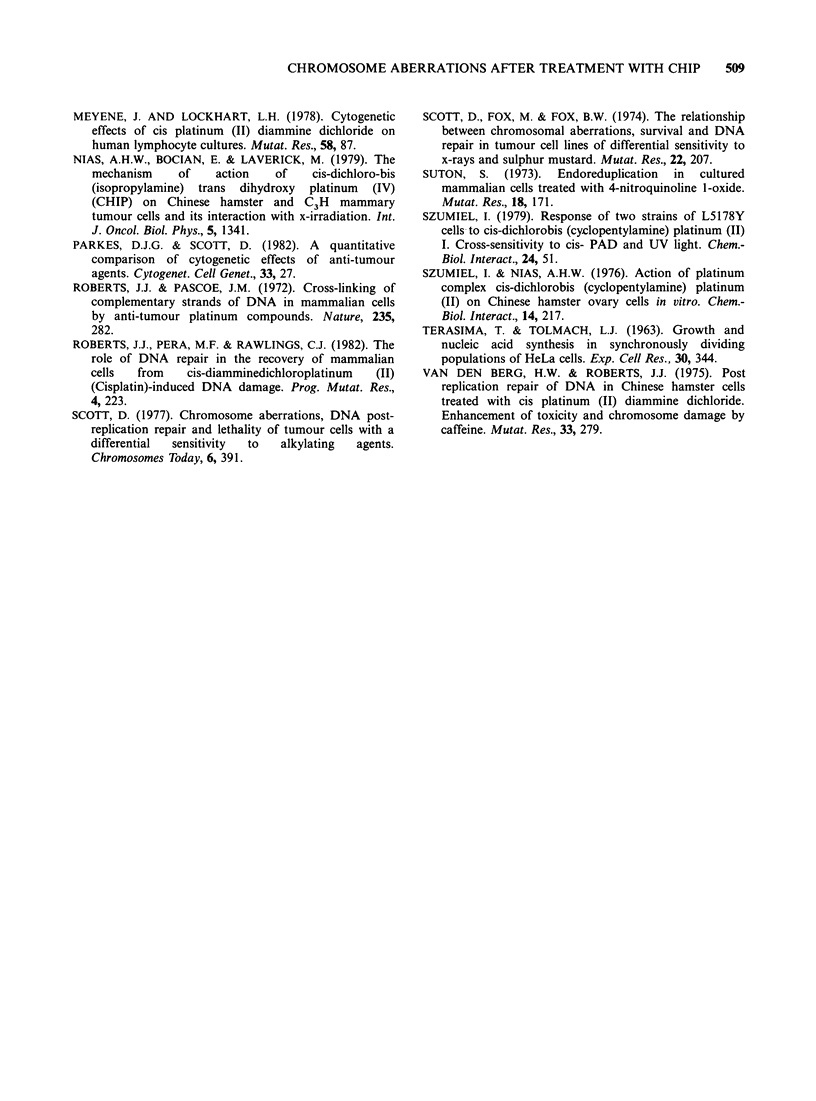# The mode of action of cis dichloro-bis (isopropylamine) trans dihydroxy platinum IV (CHIP) studied by the analysis of chromosome aberration production.

**DOI:** 10.1038/bjc.1983.80

**Published:** 1983-04

**Authors:** E. Bocian, M. Laverick, A. H. Nias

## Abstract

The induction of chromosome damage by the Platinum complex CHIP in Chinese hamster ovary (CHO) cells has been studied, together with the relationship between cell survival and aberration frequency. The type and frequency of chromosome aberrations observed in asynchronous and G1 phase treated cells indicated a similar mode of action to that of bifunctional alkylating agents. A log-linear relationship was observed between the frequency of chromatid aberrations (excluding gaps) and the level of survival after CHIP treatment, with approximately one aberration per cell corresponding to 37% survival.


Br. J. Cancer (1983), 47, 503–509

### The mode of action of *cis* dichloro-bis (isopropylamine) *trans* dihydroxy platinum IV (CHIP) studied by the analysis of chromosome aberration production

E. Bocian*, M. Laverick & A.H.W. Nias

*Richard Dimbleby Department of Cancer Research, St. Thomas's Hospital Medical School, London SE1 7EH*

**Summary** The induction of chromosome damage by the Platinum complex CHIP in Chinese hamster ovary (CHO) cells has been studied, together with the relationship between cell survival and aberration frequency. The type and frequency of chromosome aberrations observed in asynchronous and $G_1$ phase treated cells indicated a similar mode of action to that of bifunctional alkylating agents. A log-linear relationship was observed between the frequency of chromatid aberrations (excluding gaps) and the level of survival after CHIP treatment, with approximately one aberration per cell corresponding to 37% survival.

*Cis* dichloro-bis (isopropylamine) *trans* dihydroxy platinum IV—CHIP—is one of the new platinum co-ordination complexes in the group of potential antitumour agents, whose cytotoxic action has now been studied extensively. It can be compared with another platinum complex *cis*-dichloro-bis (cyclopentylamine) platinum (II), PAD, which although insoluble in water was found to have a high antitumour activity and a large therapeutic index (235), (Connors *et al.*, 1972). In contrast, CHIP is highly water soluble and this has encouraged further investigation into its mode of action.

Results from several workers indicate that DNA is the primary intracellular target for the cytotoxic action of the platinum complexes, where inter- and intra-strand crosslinks are produced (Roberts & Pascoe, 1972; Kelman *et al.*, 1977). These effects of platinum complexes on DNA point to a similar mode of action to that observed with bifunctional alkylating agents; i.e. the production of "delayed type" chromosome aberrations as a result of DNA synthesis on a damaged template (Bender *et al.*, 1974).

Studies were undertaken to examine this possibility and to understand better the mechanism underlying the cytotoxic action of CHIP. In this paper the results of the investigation of the production of chromosome aberrations by CHIP in Chinese hamster ovary (CHO) cells and the relationship of these aberrations to cell killing are presented.

*Present Address: Zaktad Radiobiologii i Ochrony Zdrowia, Instytut Badan Jadrowych, Dorodna 16, 03-195 Warszawa, Poland

Correspondence: M. Laverick

Received 15 September 1982; accepted 30 December 1982

### Materials and methods

### *Cell culture*

During the course of this work two aneuploid clones of Chinese hamster ovary (CHO) cells were used. Each had predominantly 23 chromosomes but the parent clone H had more polyploid cells than the subsequent sub-clone 10. Clone H showed slightly less sensitivity to CHIP than clone 10, with respect to the $D_q$ values of their dose-response curves ($53\,\mu g\,ml^{-1}$ and $27\,\mu g\,ml^{-1}$ respectively) although the $D_0$ values were the same ($19.4\,\mu g\,ml^{-1}$). Cells were cultured as a monolayer in disposable plastic tissue-culture flasks (Nunclon) in HEPES-buffered (25 mM) Ham's F12 medium supplemented with 15% calf serum, non-essential amino acids and 2 mM glutamine. No antibiotics were used.

### *Cell survival*

Survival of cells was determined by their colony forming ability after 5 day's growth, as described previously (Szumiel & Nias, 1976).

### *Synchrony*

Synchronization was obtained by the mitotic selection method (Terasima & Tolmach, 1963). The degree of synchrony was estimated from the measurement of the mitotic index immediately after mitotic selection. This was usually between 85–95%.

© The Macmillan Press Ltd., 1983

*Platinum complex*

*Cis* dichloro-bis (isopropylamine) *trans* dihydroxy platinum IV (CHIP) was kindly supplied by Johnson Mathey and Co. Ltd.

*Drug treatment*

In all experiments cells were exposed to CHIP in culture medium for 1 h at 37°C after they had adhered to the surface of the flask. The stock solution of drug was dissolved at a concentration of $1\,mg\,ml^{-1}$ in cold physiological saline and kept in the dark. All drug preparations were freshly prepared, immediately before each experiment. After the cells were exposed for 1 h the drug-containing medium was sucked off and replaced with fresh medium.

*Chromosome preparations and analysis*

Colcemid at a final concentration of $1\ \mu g\ ml^{-1}$ was used. Cells were hypotonized with 0.7% sodium citrate solution for 7–13 min at 37°C. Thereafter cells were fixed and washed in three changes of a 3:1 mixture of absolute methyl alcohol and glacial acetic acid, and microscopic preparations were made. Preparations were air dried and stained with 10% Giemsa solution.

All types of chromatid aberrations were scored separately and classified as chromatid and isochromatid breaks and gaps, chromatid exchanges, single fragments and chromatid interstitial deletions. An aberration was classified as a break where there was a displacement of a chromatid fragment or where the distance between two parts of chromatid arm was larger than the diameter of the chromatid. Those cells exhibiting numerous aberrations, in which an accurate analysis was too difficult, were recorded as multiaberration cells. Gaps were scored, although there is some uncertainty about their significance and they probably do not contribute to lethality.

### Results

*Chromosome aberration production by CHIP in CHO clone H cells*

An asynchronous population of CHO clone H cells was used to determine the effects of treatment with increasing concentrations of CHIP. After treatment with the drug, cells were incubated for 6 h at 37°C. Colcemid was added to the cultures during the last 2 h of the experiment, before mitotic selection. Mitotic cells harvested at this time were those treated with the drug in the middle of the S phase. (For duration of cell cycle, see Szumiel & Nias, 1976). Table I shows the frequency of aberrations in cells treated with a CHIP dose of $94\ \mu g\ ml^{-1}$ after which the surviving fraction is 0.1 (Nias *et al.*, 1979). A relatively low frequency of aberrations was produced compared with the high cell killing observed with the same dose of CHIP. Only chromatid type aberrations were observed. After the subtraction of control levels of aberrations, a linear dose-response relationship was found (Figure 1) between chromatid breaks and doses of CHIP corresponding to the exponential portion of the survival curve for clone H cells (Nias *et al.*, 1979).

To examine further the mechanism of production of aberrations by CHIP, the frequency of aberrations in the cells treated in $G_1$ and $G_2$ phase with $94\ \mu g\ ml^{-1}$ of the drug, was investigated. The effect of CHIP on the cells treated in $G_1$ phase was determined using synchronous cell populations. Mitotic cells were plated ($\sim 1$–$3 \times 10^5$ cells per flask) and after 1.5 h treated with CHIP. Colcemid was added to the cultures for the last 3 h of the experiment (i.e. between 10th and 13th h of experiment). Mitotic cells were subsequently shaken off at the first mitosis after treatment.

Asynchonous populations of cells were used to determine the effects of CHIP on cells in $G_2$ phase. Cells were treated with the drug for 1 h and with colcemid for a further hour. Thereafter, mitotic

**Table I** Frequency of chromatid aberrations at the first mitosis after treatment of CHO clone H cells with CHIP in different stages of the cell cycle

| | | | *Chromatid aberrations per cell* | | | | | |
|---|---|---|---|---|---|---|---|---|
| *Treatment* | *Cell cycle phase* | *Number of analysed mitoses* | *Breaks* | *Isochromatid breaks* | *Gaps* | *Exchanges* | *Sum of all types* | *Multi-aberration cells (%)* |
| Control | Asynchronous | 302 | 0.026 | — | 0.043 | 0.003 | 0.072 | — |
| CHIP | $G_1$ | 99 | 1.109 | 0.120 | 0.406 | 0.120 | 1.769 | 8.08 |
| $94\ \mu g\ ml^{-1}$ | Mid S | 200 | 0.240 | — | 0.155 | — | 0.400 | — |
| | $G_2$ | 146 | 0.020 | — | 0.095 | — | 0.116 | — |

**Figure 1** Relationship between the level of chromatid breaks per cell observed in asynchronous CHO clone H cells after treatment in mid S phase with increasing doses of CHIP for 1 h at 37°C.

cells were harvested immediately. Results of these experiments, i.e. the frequency of aberrations produced in $G_1$ and $G_2$ phases together with the data for mid-S cells treated with the same concentrations of CHIP are shown in Table I. In cells treated in $G_1$ phase all types of chromatid aberrations were produced, breaks and gaps being in the majority. A much higher incidence of aberrations was observed in cells exposed to CHIP in $G_1$ phase than in S phase. Many cells with numerous aberrations and exhibiting scattered chromosomes, were also noted. Cells treated in $G_2$ phase showed no difference in the level of chromatid breaks and exchanges from that found in untreated cells. Only the frequency of gaps was greater.

*Chromosome aberration production by CHIP in CHO clone 10 cells $G_1$ phase*

Three different concentrations of CHIP were used to study the effect of CHIP at two time intervals after the treatment of CHO clone 10 cells in $G_1$ phase. Results of these investigations are shown in Table II. Synchronous populations of cells were used. The experimental protocol was similar to that described for clone H except for the time of the harvest of mitotic cells to allow for differences in the time of CHIP induced mitotic delay. This was carried out both between 11 and 16h after synchronization and between 22 and 23h. Thus, for a given concentration of CHIP, replicate cultures were treated with colcemid between 11–14h and 14–16h after synchronization. and also between 22–25h and 25–28h after synchronization. The first two and the second two cell harvests were pooled and the data in Table II give the mean frequency of aberrations per cell calculated for the two periods of time, i.e. for 11–16h and 22–28h after synchronization.

From Table II it can be seen that the percentage of cells exhibiting aberrations and the frequency of aberrations per cell increase with increasing doses of CHIP. Approximately the same frequency of aberrations was produced in both the 1st and 2nd periods of mitotic cell collection after treatment with a dose of 69.7 $\mu$g ml$^{-1}$ CHIP. However, a slightly lower frequency of aberrations was found during the second period after treatment with the lowest concentration of CHIP. Although this difference may not be significant, it could be due to the appearance of a number of cells reaching their second mitosis after treatment. After the highest dose of CHIP there were no mitotic figures in these clone 10 cells during the first period of collection; during the second period many cells were found to be too heavily damaged for their mitotic figures to be analyzed. Endoreduplicated cells were also observed (~2–3%) during the second period after treatment with 69.7 and 90.9 $\mu$g ml$^{-1}$ of CHIP. In the case of clone H cells, some aberration data were obtainable after the highest dose of CHIP (Table I, Figure 1) because of the lower sensitivity of these cells.

*Correlation between chromosome aberrations and cell killing*

Figure 2 shows the survival curve for CHO clone 10 cells exposed to CHIP. The parameters of this curve are: $D_0 = 19\,\mu g\,ml^{-1}$, $D_q = 27\,\mu g\,ml^{-1}$, $n = 4.1$.

The survival of these cells, as a function of lethal aberrations (i.e. all aberrations per cell excluding gaps) found at the 1st mitosis after treatment with CHIP, is presented in Figure 3. There is a log-linear relationship between aberration frequency and cell survival, with 0.85 aberrations per cell corresponding to 37% survival.

### Discussion

*Mechanism of aberration production*

Data given in Table I show that CHIP induces all types of chromatid aberrations at the first mitosis after treatment but only in cells exposed to the drug in $G_1$ and S phases. (The gaps in $G_2$ are of doubtful significance.) These results suggest that, with respect to the production of aberrations, CHIP

**Table II** Frequency of chromatid aberrations in $G_1$ treated population of CHO clone 10 cells with different concentrations of CHIP

| CHIP treatment $\mu g\ ml^{-1}$ | Time after treatment, (h) | Number of analysed mitosis | Cells with aberrations (%) | Cells with numerous aberrations (%) | Chromatid aberrations per cell: Breaks | Isochromatid breaks | Gaps | Exchanges | Sum of all types |
|---|---|---|---|---|---|---|---|---|---|
| Control | 11-13 | 200 | 5.50 | — | 0.005 | 0.010 | 0.030 | — | 0.055 |
| 47.6 | 11-16 | 288 | 61.8 | 5.5 | 0.547 | 0.147 | 0.180 | 0.246 | 1.165 |
| | 22-28 | 258 | 48.0 | 1.5 | 0.476 | 0.224 | 0.090 | 0.125 | 1.004 |
| 69.7 | 11-16 | 158 | 69.6 | 5.7 | 0.865 | 0.174 | 0.221 | 0.362 | 1.718 |
| | 22-28 | 198 | 60.4 | 5.7 | 0.872 | 0.171 | 0.281 | 0.320 | 1.729 |
| 90.9 | 22-28 | 90 | 87.5 | 25.0 | 1.530 | 0.409 | 0.484 | 0.909 | 3.560 |

**Figure 2** Dose-survival curve obtained after treatment of CHO clone 10 cells with increasing doses of CHIP for 1 h at 37°C. Mean values are from 5 experiments except where single points are drawn (1 experiment only).

**Figure 3** Relationship between the level of chromatid aberrations per cell (all types excluding gaps) observed in CHO clone 10 cells at the 1st mitosis after treatment with CHIP and surviving fraction.

acts in a similar way to the bifunctional alkylating agents and to UV light and, therefore, falls into Class III of Bender's Classification of chemicals. These are the group of agents producing chromatid aberrations from drug-induced single strand lesions delivered in $G_1$-S phase which are expressed during S phase as a result of DNA synthesis on the damaged template and are revealed at the first mitosis thereafter. They are therefore described as "delayed-type" aberrations (Bender *et al.*, 1974).

A higher incidence of gaps in cells treated with CHIP in $G_2$ phase, as compared to the control, was obtained and this is in agreement with the findings of Bender *et al.* (1973) and Hittelman & Rao (1974) who also found that UV light and alkylating agents produced gaps in $G_2$ treated cells. The nature of the production of gaps is still not clear, but Bender *et al.* (1974) suggested in their general model of aberration production, that a single polynucleotide strand break could be manifested at metaphase, as an achromatic lesion or gap. If this model is correct, gaps produced by CHIP in $G_2$ phase cells could be a result of the operation of excision-type repair processes. Because of the uncertainty about their contribution to lethality the gap data were excluded from Figure 3.

Other platinum complexes such as *cis*-diammine dichloro platinum (II) (*cis*-PDD) and *cis*-dichlorobis (cyclopentylamine) platinum (II) (PAD) have also been found to produce chromatid aberrations of the "delayed type" (Van den Berg & Roberts, 1975; Szumiel & Nias, 1976; Meyene & Lockhart, 1978). However, Szumiel and Nias (1976) showed in the same system of CHO cells that at the first mitosis after treatment of cells in $G_1$ phase with PAD no exchanges or isochromatid breaks were produced. These were only seen at the second mitosis. The model presenting the mechanism of action of PAD on chromosomes was given by Chadwick *et al.* (1976). It implies that only single polynucleotide strand breaks opposite damaged regions are produced during the first DNA replication after treatment of cells with PAD. This means that cells are unable to excise lesions in DNA produced by PAD. According to Bender's general theory of chromatid aberration production, chromatid breaks and exchanges arise as a consequence of the second DNA replication. Thus, in cells treated with PAD, mainly gaps were seen at the first mitosis after treatment, and breaks and exchanges at the second mitosis after treatment whilst CHIP produced all types of chromatid aberrations, including isochromatid breaks and exchanges, at the first mitosis after treatment.

There is evidence for the ability of cells to repair platinum-induced lesions in DNA (Roberts *et al.*, 1982). Therefore, the reason for the differences observed in aberration production by CHIP and PAD could partly lie in the differing ability of the cells to excise the lesions induced by the two platinum complexes which itself might also be connected with the different type and/or position of the lesions produced.

Endoreduplicated cells were observed after treatment with CHIP. It is well known that as a consequence of the endoreduplication process, daughter cells frequently receive differing chromosome complements as a result of non-disjunction and multipolar divisions and this also leads to the polyploidisation of cells. It has been found that endoreduplication can also be induced in the second and subsequent generations after treatment of cells with other chemicals such as $\beta$ mercapto-ethanol and nitroquinoline-1 oxide and also with x-irradiation (Suton, 1973).

### *The frequency of chromosome aberrations and cell killing*

Although there is a well-established relationship between chromosomal aberrations and radiation-induced cell death (e.g. Dewey *et al.*, 1978), the contribution of chromosomal aberrations to cell lethality induced by chemical agents has been studied to a much lesser extent (reviewed by Scott, 1977). Platinum compounds were not included in these studies nor in the quantitative comparison of cytogenetic effects of x-rays and anti-tumour drugs by Parkes & Scott (1982). It therefore seemed of interest to analyze the relationship between the clonogenic ability of CHIP treated cells and the frequency of chromosomal aberrations.

These parameters have been determined on different cell populations; asynchronous for survival determination by cloning and synchronous ($G_1$) for analysis of chromatid aberrations. This was justifiable because of the lack of sensitivity dependence on cell age (Ackers, 1983). Such a lack of dependence was found earlier with PAD for which Szumiel & Nias (1976) also observed no age dependency of cytotoxic activity.

The data on chromatid aberration frequency were taken from Table II. CHIP induced mitotic delay is dose dependent and this was confirmed by pulse labelling the synchronous CHO cell population treated with a dose of CHIP to give a SF of 0.1 as well as from clone-size analysis for the dose of CHIP giving survival levels of 0.4 and 0.1 respectively (Ackers, 1983). It was therefore plausible to assume that the more sensitive clone 10 cells treated with the highest (90.9 $\mu g\ ml^{-1}$) dose of CHIP (SF = 0.03) did not pass their first mitosis after treatment at the time interval studied. These data on aberration frequency have been plotted with the corresponding survival data in Figure 3.

As can be seen, survival of CHO cells exposed to CHIP is log-linearly related to the chromatid aberration frequency with a level of 0.85 aberrations per cell corresponding to 37% survival. It was also found that the doses of CHIP reducing survival of both CHO clone H and clone 10 to a similar level induce similar frequencies of chromatid aberrations (Tables I and II and Figure 2—SF = 0.1 and 0.13 in CHO clone H and clone 10 respectively, with corresponding levels of aberrations per cell of 1.77 and 1.71.

A positive correlation between the lethal effects of PAD and the frequency of chromatid breaks in two mouse lymphoma L5178Y cell strains, differing in their sensitivity to PAD, was found by Szumiel (1979). A similar correlation between sensitivity to sulphur mustard and x-irradiation with chromosome aberration frequency in two Yoshida strains was also observed by Scott *et al.* (1974). On the other hand, Parkes & Scott (1982) found no correlation between the incidence of structural aberrations and the survival of diploid human fibroblasts when the effects of x-rays and four other cytotoxic agents were compared at equitoxic doses.

A log-linear relationship similar to that shown in Figure 3 between survival and chromatid aberration frequency was shown in x-irradiated CHO cells by Dewey *et al.* (1978). Their results suggested that only one aberration per cell is required for cell death and that chromatid type aberrations appeared to be as lethal as the chromosome type. So far, there are no satisfactory explanations of these observations.

Aberrations which affect only one sister chromatid would be expected to result in 50% cytologically normal daughter cells after the first mitosis. This would only be correct for cells which have one aberration per cell and therefore it would relate only to those cells treated with the lowest dose of drug used in this study. The good correlation between survival and chromatid aberration frequency may result from a delayed formation of chromosomal aberrations in cells originating from the apparently normal first generation cells. It may also be fortuitous that other lethal events, not discernible cytologically, log-linearly related to the drug dose, may increase the lethality in a manner apparently related to the aberration frequency. One such event may be that leading to interphase cell death.

### *Conclusion*

Our experiments here have confirmed that CHIP can be classified, along with other platinum co-ordination complexes and bifunctional alkylating agents, into Class III of Bender's chemical classification (Bender *et al.*, 1974). Differences observed in the type of chromatid aberrations produced by PAD and CHIP, especially at first mitosis after treatment, suggested differences of type and position of the lesions induced by the two compounds in DNA. This in turn could affect the cells' ability to excise the lesions. We have also found a log-linear relationship between survival and the chromatid aberration frequency determined at first mitosis after treatment.

The authors thank Dr. I. Szumiel for helpful discussion. This work was supported by grants from the United States National Cancer Institute and the United Kingdom Cancer Research Campaign.

### References

ACKERS, H.A. (1983). Effects of Radiosensitizing Platinum Drug on Mammalian Cells *in vitro*. *Ph.D. Thesis London University*.

BENDER, M.A., GRIGGS, H.G. & BENFORD, J.S. (1974). Mechanism of chromosomal aberration production III. Chemicals and ionizing radiation. *Mutat. Res.*, **23**, 197.

BENDER, M.A., GRIGGS, H.G. & WALKER, P.L. (1973). Mechanism of chromosomal aberration production I. Aberration induction by ultraviolet light. *Mutat. Res.*, **20**, 387.

CHADWICK, K.H., LEENHOUTS, H.P., SZUMIEL, I. & NIAS, A.H.W. (1976). An analysis of the interaction of a platinum complex and radiation with CHO cells using the molecular theory of cell survival. *Int. J. Radiat. Biol.*, **30**, 511.

CONNORS, T.A., JONES, M., ROSS, W.C., BRADDOCK, P.D., KHOKHAR, A.R. & TOBE, M.L. (1972). New platinum complexes with anti-tumour activity. *Chem.-Biol. Interact.*, **5**, 415.

DEWEY, W.C., SAPARETO, S.A. & BETTEN, D.A. (1978). Hyperthermic radiosensitization of synchronous Chinese hamster cells: relationship between lethality and chromosomal aberrations. *Radiat. Res.*, **76**, 48.

HITTELMAN, W.N. & RAO, P.N. (1974). Premature chromosome condensation II. The nature of chromosome gaps produced by alkylating agents and ultraviolet light. *Mutat. Res.*, **23**, 259.

KELMAN, A.D., PERESIE, H.J. and STONE, P.J. (1977). An analysis of modes of binding of anti-tumour platinum complexes to DNA. *J. Clin. Hematol. Oncol.*, **7**, 440.

MEYENE, J. AND LOCKHART, L.H. (1978). Cytogenetic effects of cis platinum (II) diammine dichloride on human lymphocyte cultures. *Mutat. Res.*, **58**, 87.

NIAS, A.H.W., BOCIAN, E. & LAVERICK, M. (1979). The mechanism of action of cis-dichloro-bis (isopropylamine) trans dihydroxy platinum (IV) (CHIP) on Chinese hamster and $C_3H$ mammary tumour cells and its interaction with x-irradiation. *Int. J. Oncol. Biol. Phys.*, **5**, 1341.

PARKES, D.J.G. & SCOTT, D. (1982). A quantitative comparison of cytogenetic effects of anti-tumour agents. *Cytogenet. Cell Genet.*, **33**, 27.

ROBERTS, J.J. & PASCOE, J.M. (1972). Cross-linking of complementary strands of DNA in mammalian cells by anti-tumour platinum compounds. *Nature*, **235**, 282.

ROBERTS, J.J., PERA, M.F. & RAWLINGS, C.J. (1982). The role of DNA repair in the recovery of mammalian cells from cis-diamminedichloroplatinum (II) (Cisplatin)-induced DNA damage. *Prog. Mutat. Res.*, **4**, 223.

SCOTT, D. (1977). Chromosome aberrations, DNA post-replication repair and lethality of tumour cells with a differential sensitivity to alkylating agents. *Chromosomes Today*, **6**, 391.

SCOTT, D., FOX, M. & FOX, B.W. (1974). The relationship between chromosomal aberrations, survival and DNA repair in tumour cell lines of differential sensitivity to x-rays and sulphur mustard. *Mutat. Res.*, **22**, 207.

SUTON, S. (1973). Endoreduplication in cultured mammalian cells treated with 4-nitroquinoline 1-oxide. *Mutat. Res.*, **18**, 171.

SZUMIEL, I. (1979). Response of two strains of L5178Y cells to cis-dichlorobis (cyclopentylamine) platinum (II) I. Cross-sensitivity to cis- PAD and UV light. *Chem.-Biol. Interact.*, **24**, 51.

SZUMIEL, I. & NIAS, A.H.W. (1976). Action of platinum complex cis-dichlorobis (cyclopentylamine) platinum (II) on Chinese hamster ovary cells *in vitro*. *Chem.-Biol. Interact.*, **14**, 217.

TERASIMA, T. & TOLMACH, L.J. (1963). Growth and nucleic acid synthesis in synchronously dividing populations of HeLa cells. *Exp. Cell Res.*, **30**, 344.

VAN DEN BERG, H.W. & ROBERTS, J.J. (1975). Post replication repair of DNA in Chinese hamster cells treated with cis platinum (II) diammine dichloride. Enhancement of toxicity and chromosome damage by caffeine. *Mutat. Res.*, **33**, 279.